# Comparing code-free and bespoke deep learning approaches in ophthalmology

**DOI:** 10.1007/s00417-024-06432-x

**Published:** 2024-03-06

**Authors:** Carolyn Yu Tung Wong, Ciara O’Byrne, Priyal Taribagil, Timing Liu, Fares Antaki, Pearse Andrew Keane

**Affiliations:** 1 Institute of Ophthalmology, University College London, 11-43 Bath St, London, EC1V 9EL UK; 2 Moorfields Eye Hospital NHS Foundation Trust, London, UK; 3 Faculty of Medicine, The Chinese University of Hong Kong, Hong Kong SAR, China; 4 The CHUM School of Artificial Intelligence in Healthcare, Montreal, QC Canada; 5 NIHR Moorfields Biomedical Research Centre, London, UK

**Keywords:** Machine learning, Code-free deep learning, Automated-machine learning, Artificial intelligence

## Abstract

**Aim:**

Code-free deep learning (CFDL) allows clinicians without coding expertise to build high-quality artificial intelligence (AI) models without writing code. In this review, we comprehensively review the advantages that CFDL offers over bespoke expert-designed deep learning (DL). As exemplars, we use the following tasks: (1) diabetic retinopathy screening, (2) retinal multi-disease classification, (3) surgical video classification, (4) oculomics and (5) resource management.

**Methods:**

We performed a search for studies reporting CFDL applications in ophthalmology in MEDLINE (through PubMed) from inception to June 25, 2023, using the keywords ‘autoML’ AND ‘ophthalmology’. After identifying 5 CFDL studies looking at our target tasks, we performed a subsequent search to find corresponding bespoke DL studies focused on the same tasks. Only English-written articles with full text available were included. Reviews, editorials, protocols and case reports or case series were excluded. We identified ten relevant studies for this review.

**Results:**

Overall, studies were optimistic towards CFDL’s advantages over bespoke DL in the five ophthalmological tasks. However, much of such discussions were identified to be mono-dimensional and had wide applicability gaps. High-quality assessment of better CFDL applicability over bespoke DL warrants a context-specific, weighted assessment of clinician intent, patient acceptance and cost-effectiveness. We conclude that CFDL and bespoke DL are unique in their own assets and are irreplaceable with each other. Their benefits are differentially valued on a case-to-case basis. Future studies are warranted to perform a multidimensional analysis of both techniques and to improve limitations of suboptimal dataset quality, poor applicability implications and non-regulated study designs.

**Conclusion:**

For clinicians without DL expertise and easy access to AI experts, CFDL allows the prototyping of novel clinical AI systems. CFDL models concert with bespoke models, depending on the task at hand. A multidimensional, weighted evaluation of the factors involved in the implementation of those models for a designated task is warranted.

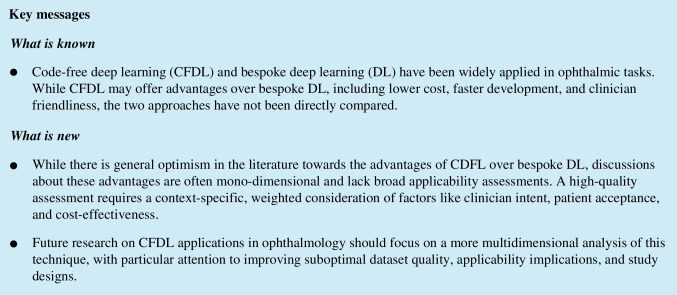

## Introduction

Building machine learning (ML) and deep learning (DL) algorithms requires technical, mathematical and engineering knowledge of artificial intelligence (AI) [1]. Hand-crafting ML or DL models can be laborious even for highly experienced AI engineers [1]. Code-free deep learning (CFDL) is a novel subtype of DL [2] that enables people without coding expertise to construct AI systems [2]. Automated machine learning (AutoML) is one form of CFDL that automates the time-consuming tasks of ML model development, including tasks of feature selection and hyperparameter optimization [3]. With commercial platforms like Google and Apple offering user-friendly, open-access interfaces for the public to develop their own CFDL models [2], there has been a lot of interest surrounding this new form of AI in the ophthalmological field.

In recent years, ophthalmologists began exploring the potential of CFDL in screening, disease diagnoses and outcome prognoses [2] and comparing their behaviour to equivalent bespoke ML/DL models designed for similar purposes [4]. CFDL has already shown strong discriminative capacities in multiple tasks using a variety of ophthalmic imaging modalities, including optical coherence tomography (OCT) scans and fundus photos [2]. Furthermore, multiple studies have reported on CFDL algorithms that have matched or even surpassed the performance of comparable bespoke DL models [5–8]. With this growing evidence supporting the potential of CFDL, it is undoubtedly changing the landscape of AI development in ophthalmology and has the potential to empower ophthalmologists with tools to develop their own algorithms.

Although CFDL is spearheading positive advancements in AI, there remain barriers that are limiting its widespread adaptation. One of these limitations is the ‘black box’ nature of those models—meaning that the model decisions can no longer be understood by humans when the model becomes sufficiently complex [9, 10]. ‘Black box’ in ML stems from the opaque process between the data input and the final derivation of outputs [11]. This is particularly prominent for CFDL, as the selection of techniques in the model’s iterative process of testing and modifying hyperparameters remains hidden [12]. In contrast, bespoke ML/DL involves experts manually choosing the architecture to build the model around and intuitively adjusting the hyperparameters [12]. Hence, it is plausible that bespoke ML/DL offers clinicians better insight into the algorithm’s inner mechanisms and a lesser black box [13]. For that reason, CFDL’s suitability in ophthalmological tasks of different natures can be debatable.

In this article, we aim to review current CFDL and traditional bespoke DL applications in ophthalmology. As exemplars, we use five important tasks in our field: (1) diabetic retinopathy screening, (2) retinal multi-disease classification, (3) surgical video classification, (4) oculomics and (5) resource management. Our goal is to explore whether CFDL can replace bespoke DL and whether ophthalmologists are approaching decisions regarding AI’s implementation in a context-aware and holistic manner.

## Methods

**Fig. 1:**
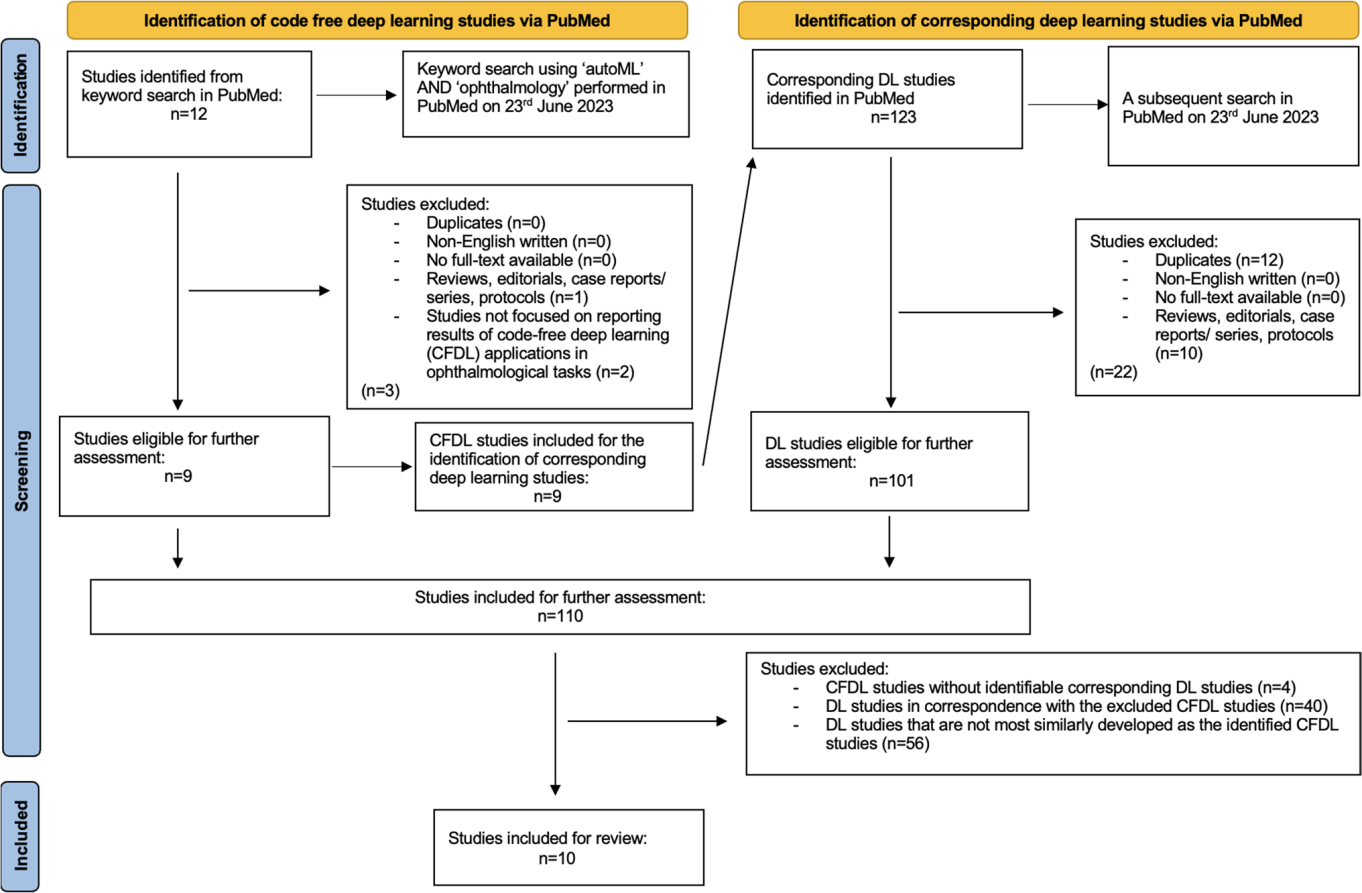
Search strategy

We performed a focused search for studies reporting CFDL applications in ophthalmology through MEDLINE/ PubMed on June 25, 2023, using the keywords ‘autoML’ AND ‘ophthalmology’. A subsequent search in PubMed on the same date was performed to find equivalent DL studies performing the same ophthalmological tasks as those in the identified CFDL studies. Only English-written articles with full text available were included. Reviews, editorials, protocols and case reports/series were excluded. We identified ten relevant studies and included them in the review. The search process and literature findings are summarised in Fig. 1 and Tables 1, 2, 3, 4, and 5 respectively.

**Table 1:** CFDL or non-CFDL guided diabetic retinopathy screening

Authors	Jacoba et al. [14]	Nunez et al. [17]
Model/software	Code-free deep learning (CFDL) models trained by Google Automated Machine Learning (AutoML) platform	Deep learning algorithm (ResNet34)
Task	Diabetic retinopathy (DR) screening	DR screening
Dataset	17,829 fundus images of 3566 eyes acquired using handheld retinal cameras in a DR screening program	32,494 images from 16,247 eyes (9778 individuals) from SMART-INDIA 1 and SMART-INDIA 2
Performance metrics	Accuracy (ACC) 97% Precision (PR) 97% Recall (RC) 97% Area under precision-recall curve (AUPRC) 0.955 F1 score 96% Sensitivity (SN) 96% Specificity (SP) 98% Positive predictive value (PPV) 96% Negative predictive value (NPV) 98%	Area under receiver operating curve (AUROC) 0.99 SN 93.86% SP 96%
Externally validated performance metrics	ACC 97% F1 score 96% SN 94% SP 97% PPV 96% NPV 96%	NR
Other means of model performance assessment	NR	NR
Findings	- The SN and SP of the algorithm on internal and external validation sets exceed the minimum diagnostic thresholds recommended for DR screening devices in the UK - The CFDL model performed comparably to published diagnostic accuracy metrics of commercial models used for DR screening, showing the feasibility of use. Yet, a comparison between CFDL’s performance and gold standard evaluations is needed	- The DL model achieved a clinically acceptable level of performance in detecting referable DR and DME from handheld, non-mydriatic retinal images in a community setting of a low-middle income country, i.e. India - Univariate and multivariate logistic regression revealed the duration of diabetes had the highest predictive significance
Limitations	- Non-randomised controlled trial (RCT) study design - Absence of investigation on verifying the actual benefits of CFDL-led screening in improving patient outcome and cost-effectiveness - Absence of direct comparison between CFDL and non-CFDL models’ screening performance trained on the same dataset	- Monoethnic dataset - Relatively small amount of input data - Subject to pre-data-input manual curation of poor-quality field images
Clinician intention	- To develop an artificial intelligence-based screening system that is easily implemented, cost-effective and inexpensive for community-wide screenings of DR, especially in low-resource areas	- To develop a scalable, cost-effective AI screening system to detect vision-threatening retinopathy in low-resource countries

**Table 2:** CFDL- or non-CFDL-guided retinal multi-disease classification

Authors	Antaki et al. [18]	Abitbol et al. [19]
Model/software	CFDL models generated by GoogleCloud AutoML Vision	DL models developed via Tensorflow
Task	Differentiation of retinal vein occlusion (RVO) from other retinal diseases, e.g. retinitis pigmentosa (RP) and retinal detachment (RD)	Differentiation of RVO from other retinal diseases, e.g. DR and sickle cell retinopathy (SCR)
Dataset	2137 UWF pseudocolor (UWF-CFP) images Images identified from the publicly available Tsukazaki Optos Public Project dataset: 472 normal, 533 RVO, 251 RP and 881 RD images	224 UWF-CFP images of patients from Creteil University with uniform distribution among classes: 65 DR (29.0%), 47 RVO (21.0%), 57 SCR (25.4%) and 55 healthy controls (24.6%)
Performance metrics	Multi-disease classification: AUPRC 0.876 SN 77.93% PPV 82.59%	RVO-specific classification: ACC 88.4% PR 77.2% AUROC 0.912 F1 score 83.3% SN 78.7% SP 91%
Externally validated performance metrics	Multi-disease classification performance: SN 79.38% SP 98.3% PPV 95%	NR
Other means of model performance assessment	NR	NR
Findings	- The CFDL models generally showed comparable performance to the bespoke deep-learning models trained on the same data sets despite some variations occurring - The CFDL models showed the feasibility to perform multi-disease classification of retinal pathologies utilizing UWF-CFP images	- The DL model was able to effectively detect and classify several retinal vascular diseases using UWF-CFP accurately and RVO was the second-best detected class among all diseases
Limitations	- Relatively small number of images - Dataset partly representative of the diversity of pathology seen in the real-life setting and may be affected by publication bias - Inadequate reporting of dataset ethnicity, causing uncertain generalisability	- Relatively small dataset - Absence of external validation - Artefacts in UWF-CFP images
Clinician intention	- To develop an algorithm with a high level of accuracy in detecting retinal pathologies and classifying various retinal diseases (e.g. RVO, RD and RP) simultaneously for further treatment and follow-up	- To develop a highly accurate system for detecting various retinal pathologies for subsequent management and specialist follow-up or to develop a tool used for screening in remote areas

**Table 3:** CFDL- or non-CFDL-guided surgical video classification

Authors	Touma et al. [20]	Yeh et al. [21]
Model/software	CFDL models trained with Google Cloud AutoML Video Intelligence Classification	DL models: VGG model and VGG16 model (convolutional neural network [CNN]–recurrent neural network [RNN] model)
Task	Classification of phases in cataract surgery	Classification of phases in cataract surgery
Dataset	144 cataract surgery videos from Cataract-21 and Cataract-101 datasets	298 cataract surgical videos routinely recorded during residency training of 12 surgeons across 6 different sites
Performance metrics	ACC 96% PR 81% RC 77.1% AUPRC 0.855 F1 score 79% SN 46.2–100.0% SP 98.0% PPV 81.0%	- Overall top 1 prediction accuracy for the VGG model is 76% (93% for top 3 accuracy) and 84% for the VGG16 model (97% for top 3 accuracy) - Microaveraged AUROC was 0.97 for the VGG model and 0.99 for the VGG16 model - Microaveraged average precision score was 0.83 for the VGG model and 0.92 for the CNN-RNN model
Externally validated performance metrics	ACC 93% PR 54.2% RC 61.1% SN 61.1% SP 96.2% PPV 54.2%	NR
Other means of model performance assessment	NR	NR
Findings	- The CFDL model performed better than DL models in the classification of surgical phases - Discriminative performance dropped when model was tested on an independent dataset	- The DL model with a CNN plus RNN architecture showed highly accurate predictions for routine steps of cataract surgery - Visualization of the gradient map was also used to view important features
Limitations	- Limited generalizability - Uncertainty on automatic segmentation and classification of surgery videos - Black-box nature	- Relatively few videos with rare steps - Uncertain level of training of surgeons in videos - Limited size and variability in the overall training set
Clinician intention	- To utilise AI to help create extensive libraries containing surgical video segments of procedures (e.g. cataract surgery) for trainees to gain access to medical knowledge at all times for better self-learning	- To develop an algorithm that recognises basic and complex activities in cataract surgery, allowing automated and detailed analyses of cataract surgical videos to pool cataract surgery experience from various surgeons for trainees seeking to improve their surgical performance from self-directed learning

**Table 4:** CFDL- or non-CFDL-guided oculomics

Authors	Korot et al. [22]	Munk et al. [23]
Model/software	CFDL models trained with Google Cloud AutoML platform	DL classifier utilising CNN
Task	Sex prediction	Sex and age prediction
Dataset	84,743 fundus photos from the UK Biobank	135,667 fundus images and 85,536 volumetric OCT scans from the Department of Ophthalmology, University Clinic Bern
Performance metrics	ACC 86.5% AUROC 0.93 SN 88.8% SP 83.6% PPV 87.3%	For sex prediction: AUROC was 0.80 for fundus images, 0.84 for OCT cross sections and 0.90 for OCT volumes
Externally validated performance metrics	ACC 78.6% SN 83.9% SP 72.2% PPV 78.2%	NR
Other means of model performance assessment	NR	NR
Findings	- The CFDL model showed robust performance for predicting sex from retinal fundus photos and was able to perform comparably to the bespoke ML model identified	- DL classifiers were able to effectively predict sex and age based on fundus images, OCT volume- and individual B-scans - Sex prediction was highest using OCT volume scans, followed by individual OCT Bscans and fundus images - Salient regions like optic disc biomarkers were also used for correct gender and age prediction as revealed by the activation map
Limitations	- Dataset not fully representative of the general UK population - Potential of patient-level overlap between the two datasets	- Model only qualitatively assessed the presence or absence of perspective biomarkers without including the quantitative evaluation
Clinician intention	- To harness the power of AI to explore and gain new insights into relationships between retinal structure and systemic pathophysiology	- To harness the power of AI to extract information and patterns which are not obvious to the human eye and explore novel biomarkers with systemic associations to cardiovascular and neurodegenerative diseases

**Table 5:** CFDL- or non-CFDL-guided resource management

Authors	Nakayama et al. [24]	Chen et al. [25]
Model/software	CFDL models trained with Amazon Forecast	ML models: XGBoost, Random Forest, Support Vector regression
Task	Forecasting of patient admission number	Forecasting no-show patient admissions
Dataset	Recorded visits (356,611) from Hospital da Universidade Federal de São Paulo	Electronic health record (EHR) retrieved from the Oregon Health and Science University with 5188 follow-up visits and 3606 new visits recorded from 7 paediatric ophthalmology departments
Performance metrics	NR	Performance of XGBoost model in follow-up patients: AUROC 0.90 Precision-recall score 0.74 SN 0.45 PPV 0.88 Performance of XGBoost model in new patients: AUROC 0.64 Precision-recall score 0.26 SN 0.14 PPV 0.25
Externally validated performance metrics	NR	NR
Other means of model performance assessment	- The accuracy metrics in daily volume prediction presented an average weighted quantile loss of 0.09, weighted absolute percentage error of 0.12 and root means a square error of 31.61	NR
Findings	- The prediction of the patient influx cases and traumas cases number at the emergency room of the ophthalmological hospital had values close to the actual values recorded in January 2020 visits and traumas cases	- The ML models trained with EHR were able to effectively predict no-shows in paediatric ophthalmology departments - The prediction of no-shows was more accurate in follow-up patients than those of new visits with the availability of longitudinal data - Number of previous visits and days between visits were important features
Limitations	- Prediction carried out in the pre-pandemic period of COVID-19 causing uncertain results - Dataset quality harmed by incomplete medical records	- Single-centre study - Uncertain model generalizability to other subspecialties - Feature importance analyses failed to give the exact effect of feature to predictions
Clinician intention	- To develop an AI system to provide a smart and effective estimation of emergency patient volume for better planning of staff and hospital resources in advance	- To develop an AI system to predict no-shows to help better adjust resources for the development of better overbooking strategies

## Results

### Diabetic retinopathy screening

A CFDL diabetic retinopathy (DR) screening algorithm was developed by Jacoba et al. [14] using 16,681 handheld camera images acquired from a local DR screening programme. The resultant model detected referable DR with a high accuracy (ACC) (above 90%). The ACC and F1 score remained at high levels when the model was internally and externally validated (ACC = 97% and F1 score = 96%; ACC = 97% and F1 score = 96%, respectively). It was claimed that the CFDL model was likely to meet the regulatory performance threshold for AI systems after the CFDL model had been compared with the performance of commercial AI systems reported in the US Food and Drug Administration (FDA)-approved documents [15]. Furthermore, all reported values of model sensitivity (SN) and specificity (SP) (both internally and externally validated values) were found to surpass the minimum diagnostic thresholds recommended for DR screening devices in the UK [16]. CFDL was suggested to be helpful for improving healthcare accessibility. However, the study suffered from limitations of a non-clinical-trial design, training data paucity, the absence of a side-by-side comparison to bespoke DL models and the inability to demonstrate the clinical effectiveness of the developed CFDL model [14].

A corresponding study using bespoke DL models on the same dataset as Jacoba et al. could not be found. We therefore compared it to another study from India reporting on a bespoke DL model that was developed by Nunez et al. [17] on 32,494 handheld camera images retrieved from two local DR screening campaigns (SMART-INDIA 1 and SMART-INDIA 2). The bespoke DL model achieved an area under the receiver operating curve (AUROC), SN and SP of 0.99, 93.86% and 96% respectively for referable DR detections. Yet, external validation had not been performed. It was proposed that the model could serve as a useful tool for helping policymakers establish scalable and cost-effective DR screening programmes in the community. However, the result findings were limited by the monoethnic and small-sized training dataset, as well as the lack of external validation [17].

### Retinal multi-disease classification

Antaki et al. [18] and Abitbol et al. [19] shared a similar interest in designing automated systems performing multi-retinal disease classifications. Antaki et al. [18] utilised 2137 ultra-widefield pseudocolor fundus photographs (UWF CFP) from a publicly available dataset to train CFDL classifiers. The resultant multi-disease classifier achieved an area under precision-recall curve (AUPRC), SN and positive predictive value (PPV) of 0.876, 77.93% and 82.59% respectively for the detection of retinal vein occlusion (RVO), retinitis pigmentosa and retinal detachment. The SN and PPV were relatively maintained at 79.38% and 95.00% respectively when the model was externally validated. The CFDL model was deemed a feasible solution for the task. However, the finite amount of input photographs and partial representativeness of the dataset to the real-world population were regarded as challenges for the model to be implemented [18].

The DL model in Abitbol et al.’s [19] study was constructed using 224 UWF CFPs collected from a hospital’s records and was intended for the delineation of RVO from other retinal vascular diseases (e.g. DR and sickle cell retinopathy (SCR)). The developed four-class classifier was able to detect RVO at a per-class AUROC and ACC of 0.912 and 88.4% respectively. SP for all four classes reached more than 90% and SN for both SCR and RVO identifications were stated to be high enough for efficient screenings (94.7% and 78.7% respectively). The multi-class model was generally regarded as an effective tool in performing multi-retinal vascular disease classification. Yet, several model shortcomings, such as the small training dataset and the lack of external validation, were noted [19].

### Surgical video classification

Touma et al. [20] developed a CFDL system for the classification of surgical phases in pre-recorded cataract surgery videos and planned to utilise the system for the creation of a surgical video library. Two publicly available datasets (122 videos) were used to train the model. The resultant model was able to classify with an AUPRC, ACC and SP of 0.855, 96.0% and 98% respectively in the internal dataset. When externally tested, the algorithm’s ACC and SP dropped slightly to 93% and 96.2% respectively. The CFDL model was claimed to perform better than bespoke models derived by AI experts. However, Touma et al. [20] revealed that the model’s limited generalisability and explainability were likely to impede the model’s implementation.

Similarly, Yeh et al. [21] used 298 cataract surgical videos recorded during the residency training of 12 surgeons to build traditional DL models for the classification of surgical phases in cataract surgical videos. The best-performing DL model was able to achieve an ACC of 84%, AUROC of 0.99 and a precision of 0.92. It was concluded that DL was highly accurate in the classification. However, more training samples were believed to be needed in future studies [21].

### Oculomics

Both Korot et al. [22] and Munk et al. [23] designed algorithms that predict sex from fundus photographs. Korot et al. utilised 175,825 fundus photos from the UK Biobank dataset to train a CFDL model. The resultant algorithm was able to predict with an AUROC, ACC, SN, SP and PPV of 0.93, 86.5%, 88.8%, 83.6% and 87.3% respectively. The ACC, SN, SP and PPV dropped to 78.6%, 83.9%, 72.2% and 78.2% respectively when the model was externally validated. The foveal region was found to be a salient feature for the model’s predictions. In summary, the CFDL was proven to be a robust framework for predicting sex. However, the algorithm suffered from limitations related to the uncertain representativeness of the training dataset to the real-world population and the unclear clinical usefulness of the algorithm [22].

Munk et al. [23] developed a similar traditional DL classifier that predicts sex from fundus and OCT images. It was revealed that the model had an AUROC of 0.80 for predictions made with fundus image information, 0.84 for predictions made with OCT cross-section images and 0.90 for predictions made with OCT volumes. Optic disc biomarkers were also revealed to be salient information used for the sex and age prediction [23].

### Resource management

CFDL and bespoke DL technologies were also used to forecast hospital admissions for resource management purposes in ophthalmological departments [24, 25]. Nakayam et al. [24] utilised 356,611 visit records documented from January 01, 2014, to December 31, 2019, at the Hospital da Universidade Federal de São Paulo to train a CFDL model for forecasting emergency patient volumes in January 2020. It was found that predictions of emergency patient volume and trauma cases were close to the actual volumes recorded in January 2020. The accuracy metrics in daily volume prediction presented an average weighted quantile loss of 0.09, weighted absolute percentage error of 0.12 and root means a square error of 31.61 [24].

Similarly, Chen et al. [25] developed a DL model to forecast ‘no-show’ patients at a paediatric ophthalmic hospital. The XGBoost model achieved an AUROC of 0.90, precision-recall (PR) score of 0.74, SN of 45% and PPV of 88% in predicting ‘no-shows’ for follow-up patients. AUROC, PR score, SN and PPV were 0.64, 0.26, 14% and 0.25 respectively for the prediction of the ‘no-show’ in new patients. It was concluded that the prediction of no-shows was more accurate in follow-up patients than those new ones [25].

## Discussion

For clinicians without DL expertise, and without easy access to experts in this area, CFDL can allow them to prototype novel clinical AI systems. At the same time, for AI experts, CFDL can potentially make the process of training models easier by accelerating the model development pipeline. From our review, it is clear from the studies that CFDL has been showing a promising horizon in multiple ophthalmological tasks including DR screening, multi-retinal disease differentiation, surgical video classification, oculomics research and resource management.

Most of the studies we reviewed were hopeful for future integrations of CFDL into different practice areas [14, 18, 20, 22, 24]. However, we note that positive conclusions drawn on CFDL’s benefits were largely based on the system-derived performance results [14, 18, 20, 22, 24]. Not all CFDL algorithms had undergone further comparison to bespoke DL to prove their unique value and benefits. Furthermore, discussions of CFDL were mostly done mono-dimensionally, seldomly discussing other implementation demands of AI, such as acceptance and applicability.

### The need for publicly available datasets for external validation

An important practice in ensuring the broad applicability of AI systems is external validation [26]. It is a vital step in the development of viable AI-powered medical decision-support systems [27]. Internal validation alone is insufficient to prove the models’ ability to maintain their performance in contexts that are different from those from which the training data was obtained [28, 29]. Often, the testing contexts in internal validation are not sufficiently different from the training contexts (e.g. data attributes), and as such, the validated model may be prone to failing generalizability in settings with distinct data contexts (i.e. data shifts) [26]. Many variables, including imaging equipment, ethnic distribution and disease manifestations in the deployment setting may result in model performance drops upon deployment [26, 30]. Thus, assertions about the applicability of CFDL models may be overstated.

To ensure the robustness of models, it is generally recommended for external validation datasets to have limited overlap with the training set [26, 31, 32]. As such, the availability of free, open-access big data sets will be important to externally validate AI models in general and CFDL model in particular [32, 33]. These open-access datasets can save researchers the cost, time and effort of manually combining and cleansing local data from various distinctive sources [32, 33]. Moreover, these datasets that span a diverse variety of populations, settings and case mix variations add to the rigorousness of the validation approach [32, 33].

### Systematic approach to model’s decision-making

When it comes to opting for an AI model for a certain task, it is unarguable that the chosen algorithm should be the best candidate for the task. In other words, it should prove its value by showing the superior task-specific advantages it offers over other AI counterparts. Hence, conclusions regarding the beneficial use of CFDL can only be drawn when it has been holistically compared to traditional DL in the task of interest. It is most accurate for ophthalmologists attempting to compare between CFDL and traditional DL’s fittingness for a task to take into account both model performance and implementation considerations. Implementation considerations include the developer’s intentions, user acceptance and cost-effectiveness. However, since trade-offs tend to exist between the different considerations [34], it is imperative that ophthalmologists weigh their relative importance and identify the model that has achieved a fine balance between the factors for the specific context. Future investigative discussions of AI are encouraged to be conducted multidimensionally to better display the model’s context-aligning benefits.

### Developer intention

Uncovering the clinician’s ultimate goal is a crucial first step for assessing the fittingness of CFDL in a specific task. In DR screening, it is evident that developers’ objectives were to find a low-cost tool to cover for ophthalmologists in community screenings [14, 17, 35]. Cost is an important consideration in this screening context, especially since the issue of limited public funding reserved for screening projects had been identified by the developers [35]. For multi-retinal-disease classification, the authors aimed to utilise the automated systems for making clinical diagnostic decisions [18, 19]. The developers were seen emphasising the model’s precision [18, 19]. Precision, in this context, is a model’s reliability in producing clinically correct diagnoses, considering plausible concerns of patient health being potentially harmed by inaccurate decisions [36].

Model interpretability is key for fostering the trustworthiness and reliability of an AI system as it opens the portal for ophthalmologists to reason with the algorithm’s operational logic and ascertain clinical justifiability within an algorithm [37, 38]. Hence, model interpretability is considered a significant model quality in the clinical diagnostic context. The knowledge of the developer’s intentions encourages a better understanding of the model qualities for successful AI integration into clinical practice with minimal clinician rejection. Such an awareness of developer intentions can be exploited to screen out CFDL as a beneficial candidate in incompatible ophthalmological tasks. For example, poorly interpretable CFDL can be ruled out as a beneficial candidate for the multi-retinal-disease diagnostic task.

### Patient acceptance

The next step in the suitability evaluation of CFDL involves the acknowledgment of the patient’s acceptance. Knowledge of patients’ concerns and attitudes towards AI’s participation in their management pathway helps to ensure the smooth implementation of the model and avoid the use of CFDL in those scenarios that involve patients’ opposition to certain qualities in CFDL. Due to the absence of patient attitude information in the CFDL studies [14, 18, 20, 22, 24], additional questionnaire studies on patient attitude were surveyed. Uncertain model reliability associated with poor model interpretability (i.e. black box) was found to be one of the greatest concerns patients have towards the use of clinical AI [39]. Interestingly, reluctance towards AI uses was expressed only when inadequately interpretable AI models were to proactively take part in high-stake decision-making [39]. However, a welcoming attitude towards AI was discerned when AI was to be utilised in low-risk settings [40]. Patients deemed the unsatisfactory model interpretability situation less worrisome as long as the ambiguous model actions play no part in direct patient management and are not empowered to potentially inflict harm on patients’ well-being [39].

In addition to patient acceptance, regulatory clearance of any AI model, whether CFDL or bespoke, remains a significant challenge. Realistically, CFDL models are best suited for non-clinical, potential use cases that do not require approval as a medical device. For example, CFDL can be used for post hoc analysis of clinical trial data, prototyping of AI system development, and development of AI systems for clinical trial feasibility planning and pre-screening.

Ensuring data privacy is also important, particularly for CFDL models since clinicians would typically upload datasets for training and testing on company-hosted websites to build models. Clinicians might not always be fully aware of how their data is stored, processed or potentially shared within these platforms. Therefore, delegating to legal regulations may assist CFDL users to safeguard their data from a legal standpoint (e.g. confidentiality agreements with AI firms on privacy issues).

### Cost factor

Cost is an integral element to pay attention to in the assessment of CFDL’s compatibility with the task nature. Operational cost and cost-effectiveness are two important concepts in the cost domain. Operational cost is a useful indicator to assist tasks with clear ‘low cost’ objectives, like DR screening, to locate potentially cost-beneficial tools on the superficial level. With previous evidence proving CFDL’s capability of processing up to 35,000 images with less than US$100 needed [13], CFDL is disposed to offer low-cost options that support the full ML workflow [41]. Yet, in reality, model cost extends beyond operational costs [42]. Hence, cost-effectiveness is a more accurate representation of the cost-beneficial attribute of an AI tool. By calculating the cost-effectiveness with the proposed formula of willingness to pay (WTP) × change in quality-adjusted life years (QALYs) − change in cost [43], an AI tool is better certified to provide long-term cost-saving benefits. The authenticity of the cost-friendly qualities in CFDL can also be validated.

### Redefining opportunities with CFDL

In light of the limited information available, a multidimensional analysis of how CFDL fits in the tasks of ophthalmological training, oculomics research and resource management is not possible. Future model studies on such tasks, as well as the previously discussed screening and diagnostic tasks, are encouraged to incorporate investigations of task intention, patient opinion and cost expectation. It can only be concluded that CFDL opens new doors of opportunity for ophthalmological training, oculomics research and resource management. CFDL may also enable the creation of surgical video libraries for trainees’ self-learning given CFDL’s ability to process vast amounts of data in a computationally less expensive manner than traditional DL [20]. As for oculomics research, CFDL may offer benefits in the early research stages, especially when there’s a minimal guarantee of results. CFDL could provide ophthalmologists with a cost-friendly platform to boldly test out their hypotheses in initial research stages without having to bear heavy financial burdens from model development. As demonstrated by Yeh et al. [21] and Munk et al. [23], model interpretability tools like saliency maps and the What-If tool could help keep an eye on the clinical relevance and plausibility of CFDL-identified novel ocular biomarkers. With more evidence amassed from CFDL analyses on the potential biomarkers, it becomes incentivising to perform DL studies to verify the legitimacy of the CFDL-discovered novel biomarkers. This is because investments in the construction of traditional DL models for mass data analyses tend to be financially dissuasive in the face of little proof of success [44, 45]. In terms of resource management, CFDL was seen making accurate patient admission forecasts at an ophthalmology department and was believed to favour hospital resource management [24]. Taking into account the fact that future admission predictions are liable to high levels of fluctuation in an ever-changing clinical environment [24], like the hit of a pandemic, readily accessible CFDL can be exploited to guide resource planning, e.g. staff and operation theatre in advance with its rough estimations of patient volume [24].

To summarise, CFDL should be evaluated multidimensionally on a case-by-case basis in order to draw conclusions regarding its helpful impact. We did not emphasise performance considerations in our evaluation since comparisons between CFDL and bespoke DL models are prone to bias, especially when different datasets are used to create models for the same task. Furthermore, it is more practical to compare the model’s diagnostic performance to current clinically established gold standards of diagnoses in order to provide evidence supporting the use of AI in current clinical practices, especially since the majority of CFDL and bespoke DL models have achieved high accuracy (80–90%). Therefore, an exact value-to-value comparison in model performance measures has limited implications in the decision-making of a model for the task, given the model’s sensitivity to vary with the dataset and training dynamics [46, 47].

## Limitations

Our model-to-model comparison per task is subject to biases because of the different datasets used to develop the CFDL and bespoke models, despite attempts to find models developed using similar datasets for each task. In addition, external validation had not been routinely performed across studies to verify claims of model robustness, making the reports on model performance liable to biases and attempts of objective model-to-model performance comparisons challenging. Besides, even if the studies had externally validated their models’ performance, it is arguable whether the external validation carried was effective in proving the model’s ability to withstand adversarial attacks in potential deployments to real-world settings in view of the uncertainties towards the characteristic differences between the external dataset and training dataset.

Furthermore, regardless of patients’ relative acceptance of uninterpretable predictions in certain settings, ‘black-box’ remains a persistent challenge for patients and ophthalmologists to fully embrace AI’s entry to an expertise that so heavily relies on an evidence-based practice [48, 49]. Therefore, in reality, ‘black-box’ of all sizes will more or less face the same cynicism in all application settings, unless the ‘black-box’ is resolved. Finally, most of the reviewed literature were single-centre studies of observational nature and were found to have struggled with relatively small dataset sizes and class imbalance issues in their training dataset. They could contribute to more biases in the analyses. Future FDA-regulated randomised controlled trials to study CFDL and bespoke DL performance in various tasks are warranted. Multi-centre collaborative efforts to create benchmark datasets of larger sizes and diverse patient characteristics are also needed to improve the training datasets’ representativeness to real-world situations and better guarantee the model’s maintenance in performance when deployed.

## Conclusion

CFDL has exhibited exciting results comparable to bespoke DL models, alongside substantial advantages in a variety of ophthalmological tasks like DR screening, multi-retinal disease classification, surgical video classification, oculomics research, and resource management. Our discussion highlighted the need to conduct a comprehensive assessment of both implementation (model cost, objectives and acceptability) and performance factors, before deciding on the best model for the job. The main takeaway from this paper is that CFDL is unlikely to replace traditional DL in ophthalmology, and their worth varies depending on the task. Both models can perhaps be utilised concurrently at different phases of a given task.

## Abbreviations

AI: Artificial intelligence
DL: Deep learning
ML: Machine learning
CFDL: Code-free deep learning
AutoML: Automated machine learning
OCT: Optical coherence tomography
DR: Diabetic retinopathy
ACC: Accuracy
SN: Sensitivity
SP: Specificity
AUROC: Area under receiver operating curve
UWF CFP: Ultra-widefield pseudocolor fundus photographs
AUPRC: Area under precision-recall curve
PPV: Positive predictive value
RVO: Retinal vein occlusion
SCR: Sickle cell retinopathy
PR: Precision-recall
WTP: Willingness to pay
QALYs: Quality-adjusted life years
FDA: US Food and Drug Administration
